# Dedicated inserter facilitates immediate postpartum IUD insertion

**DOI:** 10.9745/GHSP-D-13-00151

**Published:** 2013-11-14

**Authors:** Paul D Blumenthal, Maxine Eber, Jyoti Vajpayee

**Affiliations:** a Population Services International, Washington, DC, USA; b Stanford University, Palo Alto, CA, USA

## Abstract

A specially designed inserter aims at facilitating IUD insertion within 10 minutes to 48 hours after delivery during the postpartum period when demand for, and health benefits of, contraception are high.

Short birth-to-pregnancy intervals are associated with poor perinatal and maternal health outcomes.^1^^–^^4^ Recent data point to a high level of unmet need for family planning among women in the first year following delivery.^5^ Improving access to family planning information and a range of contraceptive choices immediately following delivery can result in higher contraceptive uptake and help address unmet need among women who might not otherwise access such services.

Immediate postpartum intrauterine device (PPIUD) insertions within 10 minutes to 48 hours after delivery can reduce barriers to postpartum contraceptive use by offering women a highly effective, safe family planning method when it is most convenient to them.

A dedicated PPIUD inserter is currently not available. As a workaround, providers use IUDs packaged for interval insertions (insertions performed postabortion or any time after 6 weeks postpartum), which requires them to remove the IUD from the inserter sleeve with forceps before placing it at the uterine fundus. However, appropriate forceps may not always be available, and a series of specialized maneuvers are required for this insertion technique. Further, the string used in conventional IUD inserters is too short to be visible after PPIUD insertion.

Population Services International (PSI), in collaboration with the Stanford Program for International Reproductive Education and Services (SPIRES) and Pregna International Ltd., has created a simple, inexpensive inserter designed specifically for PPIUDs (see Figure).

**Figure:**
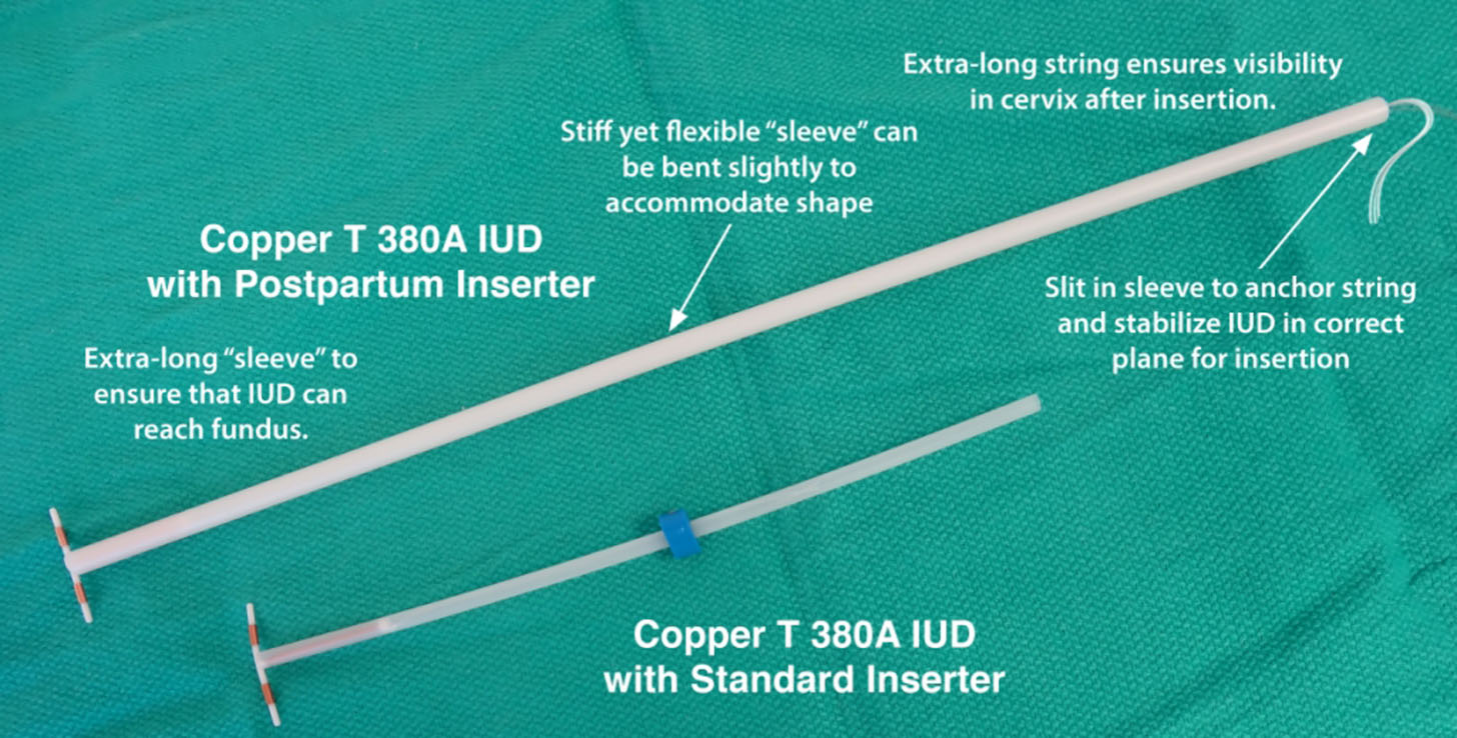
Characteristics of the New Postpartum IUD Inserter

The new inserter:

- Eliminates the need for specialized instruments such as forceps and allows for a standardized, easy-to-learn technique that mimics interval insertion
- Is made from sturdy yet bendable plastic that can accommodate the shape of the postpartum uterus
- Comes preloaded in the insertion sleeve so there is no need for manipulation, thereby reducing the opportunity for contamination and infection
- Does not require the provider to put his or her hand in the woman's vagina to insert the IUD, further reducing infection risk
- Has a longer insertion sleeve to ensure that the IUD can reach the fundus easily
- Has a longer string that is visible following a postpartum insertion
- As a dedicated product, could improve acceptability among providers of postpartum IUD provision

With seed funding from “Saving Lives at Birth: A Grand Challenge for Development,” PSI will collaborate with the Federation of Obstetric and Gynaecological Societies of India and SPIRES to conduct a proof-of-concept study followed by a clinical trial in 2 public-sector hospitals in India. The study will explore acceptability of the new inserter (provider/consumer comfort, satisfaction, and confidence), convenience, expulsion rates, and the training time required to achieve provider competency. Pregna International will provide the IUD inserters free-of-charge for the study.

For more information about the PPIUD inserter, visit the Postpartum Family Planning Toolkit at http://www.k4health.org/toolkits/ppfp.
